# Housing and management factors and breed predisposition for haemorrhagic bowel syndrome in swine

**DOI:** 10.1186/s40813-023-00340-y

**Published:** 2023-10-11

**Authors:** Fabienne Holenweger, Gertraud Schüpbach, Andreas Hofer, Xaver Sidler, Alexander Grahofer

**Affiliations:** 1 Clinic for Swine Department for Clinical Veterinary Medicine, Vetsuisse Faculty, University of Bern, Bremgartenstrasse 109a, Bern, CH-3012 Switzerland; 2 Veterninary Publich Health Institute, Department of Clinical Research and Veterinary Public Health, Vetsuisse Faculty, University of Bern, Schwarzenburgstrasse 161, Liebefeld, 3097 Switzerland; 3 SUISAG, Allmend 10, 6204 Sempach, Switzerland; 4 Division of Swine Medicine, Department of Farm Animals, Vetsuisse Faculty, University of Zurich, Winterthurerstrasse 260, Zurich, 8057 Switzerland

**Keywords:** Sudden death, Pig, Risk factor, Environment, Abdominal distension

## Abstract

**Background:**

Haemorrhagic bowel syndrome (HBS) is a sporadically occurring disorder characterized by sudden death in pigs in combination with a pale and bloated carcass with no prior signs of disease. Most often HBS is affecting fattening pigs. Due to the good general health and performance before death as well as the time point of disease shortly prior to slaughter, this syndrome means a significant economic impact for the farm and is a major animal welfare concern. Furthermore, the cause or the causing agents have not yet been identified even though it is a worldwide known problem. The aim of this study was to detect possible risk factors for the occurrence of HBS with the focus on risk factors on herd level.

**Results:**

Management and feeding strategies of 97 Swiss fattening herds with high and low HBS incidence were assessed and examined to identify risk factors for the disease. Having only pigs sired by the PREMO® breed in the herd showed to be a significant risk factor for HBS (Odds Ratio (OR) = 147) as compared to having other breeds or a mixture of multiple breeds. Furthermore, pigs from two or more origins per batch compared to having only one origin per batch significantly increased the disease risk (OR = 52). Farms with 1 decimetre greater feeding place width per finisher pig have a lower HBS incidence (OR = 0.07). The frequency of cleaning of the distribution pipes (split up into categories, e.g. once a month) was associated with being a HBS case farm (p < 0.05).

**Conclusion:**

The four factors identified in this study for the occurrence of HBS represent different aspects of the environment and management. This leads to the assumption that it is a multifactorial syndrome and a thorough examination of each herd individually is necessary to mitigate disease risk. This study suggests that part of the susceptibility to HBS is genetically determined. The reduction of HBS in the herd should be the main objective to improve the economic status of the herd and improve animal welfare.

## Background

Haemorrhagic bowel syndrome (HBS) can be the cause of sudden death in pigs and is a known problem in pig production worldwide. However, prevalence data about HBS are very rare. An overall prevalence of HBS between 0.1% and 7% was detected in the USA [1], whereas the estimated prevalence in Swiss pig farms was approximately 1% (Sidler, personal communication 2020). However, the within herd prevalence during outbreaks can vary between 19.3% and 45.5% [2]. Necropsy data from Switzerland revealed HBS in 2.7% of examined pigs [3]. The syndrome is most commonly affecting finisher pigs between four to six months of age [4, 5]. The sudden death of finisher pigs has a significant economic impact because the majority of the production costs for those pigs has already been invested and slaughter of the pigs would have been timely. Since the general health and fatting performance of the effected pigs before sudden death are within the reference values, a good financial output at slaughter could have been expected [4, 6, 7]. Stomach filled with fresh food in diseased pigs supports the assumption that the pigs were in a good general condition with an appropriate feed intake before the peracute death. Until today, the understanding of the aetiology and pathogenesis of this syndrome is still lacking [4, 8]. Microbiological investigations of faecal sample as well as ingesta have failed to identify a specific infectious agent associated with HBS and no clinical signs such as diarrhoea or reduced general condition can be linked to the syndrome [6, 7]. A final diagnosis of HBS can be established after ruling out other causes of gastrointestinal haemorrhage and sudden death such as gastric ulcers, porcine proliferative enteropathy, salmonellosis or swine dysentery [1]. However, farmers often use HBS as a suspected diagnosis, when no other causes of sudden death with distended abdomen seem likely or HBS is already a known problem on farm [9]. Furthermore, it is largely accepted amongst veterinarians and farmers that the carcasses of HBS dead pigs show pallor and a severely distended abdomen [3, 4, 9–11]. A clear definition of the presentation of HBS is not yet proven. However, the typical findings during necropsy, such as red intestines with or without volvulus of the mesentery or intestine in varying degrees and directions, is described in several studies [3, 9, 12]. However, intestinal volvulus is not always apparent in cases of HBS at necropsy [3, 9]. In one study, only in 56% of the confirmed HBS cases of 436 pigs, intestinal volvulus was detected [3]. These findings are supported from pressure measurements immediately after death, which showed that pigs can die from hypovolemic shock due to excessively high intraabdominal pressure in the absence of intestinal torsion [13]. Therefore, an infectious aetiology cannot be ruled out [1, 3–5, 9].

Regarding the treatment of pigs suffering from HBS little is known, most likely due to the peracute death without prior clinical signs. However, if affected pigs are continuously monitored, it can be seen, that the animals are reluctant to move and are increasingly vocalising, followed by signs of mouth breathing and recumbency [1]. Most often death occurs within 30 to 45 min of the onset of clinical signs [14]. Therefore, to avoid sudden death of pigs due to HBS several studies have been conducted to describe possible non-infectious and infectious factors [1, 3, 4, 6, 9, 14]. It is assumed that HBS occurs more frequently in liquid feeding systems in comparison with dry feeding [3, 14]. According to a report from South Africa *Clostridium perfringens* was isolated from 40% of submitted intestinal mucosal scrapings of grower pigs with HBS [14]. Furthermore, the season was described as a risk factor by several authors, having a higher average of cases in spring in Switzerland [3], compared to a higher percentage of HBS cases in summer in South Africa [4].However, cases of HBS also occur during the winter season and/or when a dry feeding system is in use [6]. Using whey in the liquid feeding systems is one of the most discussed risk factors for HBS, with authors proposing a maximum of 20% of whey in the daily ration [2, 15]. Focusing on feed related risk factors, a French study [8] found that the use of non-justified antimicrobial treatment as a routine treatment leads to a chronic dysbacteriosis and therefore increases the risk for HBS. As an alternative for antimicrobials the usage of organic acids in feed is promoted to hinder a bacterial overgrowth in the intestinal tract [8, 9]. A study from Switzerland [3] found a higher number of yeasts in the ileum, caecum and colon of HBS pigs at necropsy compared to other suddenly dead pigs. Furthermore, female pigs were more often affected by HBS in said study, which stands opposing to the findings of a study from Great Britain [16]. Consistent eating behaviour as well as square footage available per pig, standard growing environment and predictable behaviours might be considered as protective factors [5]. A prospective study in South Africa [7] proposed as working hypothesis that *Lawsonia intracellularis* in its acute form is the causative agent of HBS, and as alternative hypothesis that *Lawsonia intracellularis* is not the sole cause of HBS. However, the bacterium was not found to be the cause of death. Rather, in the before mentioned study, *Clostridium perfringens* proofed as the most likely cause of HBS for the examined farms. All these findings indicate that there might be a number of potential risk factors for HBS warranting further and more detailed analysis.

The aim of this study was to identify potential risk factors for the occurrence of HBS in Swiss fatting farms focusing on the environment and the management. Identification of such risk factors would enable implementing strategies to reduce the prevalence of HBS on farms, and thereby improve animal health and welfare as well as the economics of pig production.

## Results

In total, the questionnaire and the herd investigation were conducted on 97 Swiss pig herds. Three herds had to be excluded, because the investigation could not be conducted due to COVID-19 related restrictions and not responding and/or cancelling shortly before the farm investigation. Farm investigations occurred between September 2021 and October 2022 by the principal investigator with no preference of season or time of day. Overall, 49.5% (n = 48) were classified as case herds whereas 50.5% (n = 49) were classified as control herds, according to previously defined criteria. The majority (n = 85) of herds were fattening herds with exclusively grower and finishing pigs and only a few were closed herds in the case and control population respectively (n = 12).

### Descriptive statistics

**Fig. 1:**
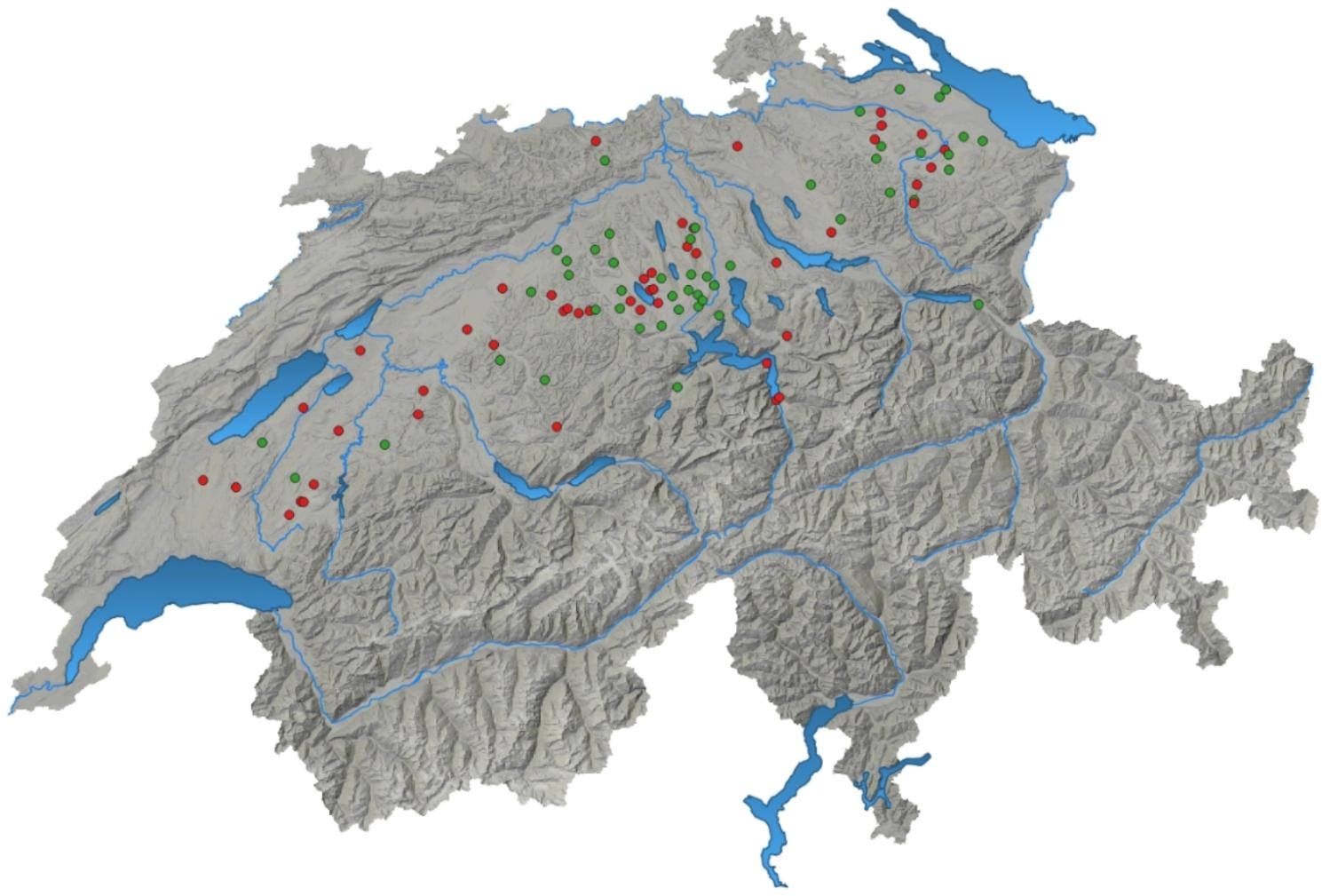
Distribution of investigated case (red) and control (green) farms in this study. This figure is based on data of 48 case and 49 control herds

**Fig. 2:**
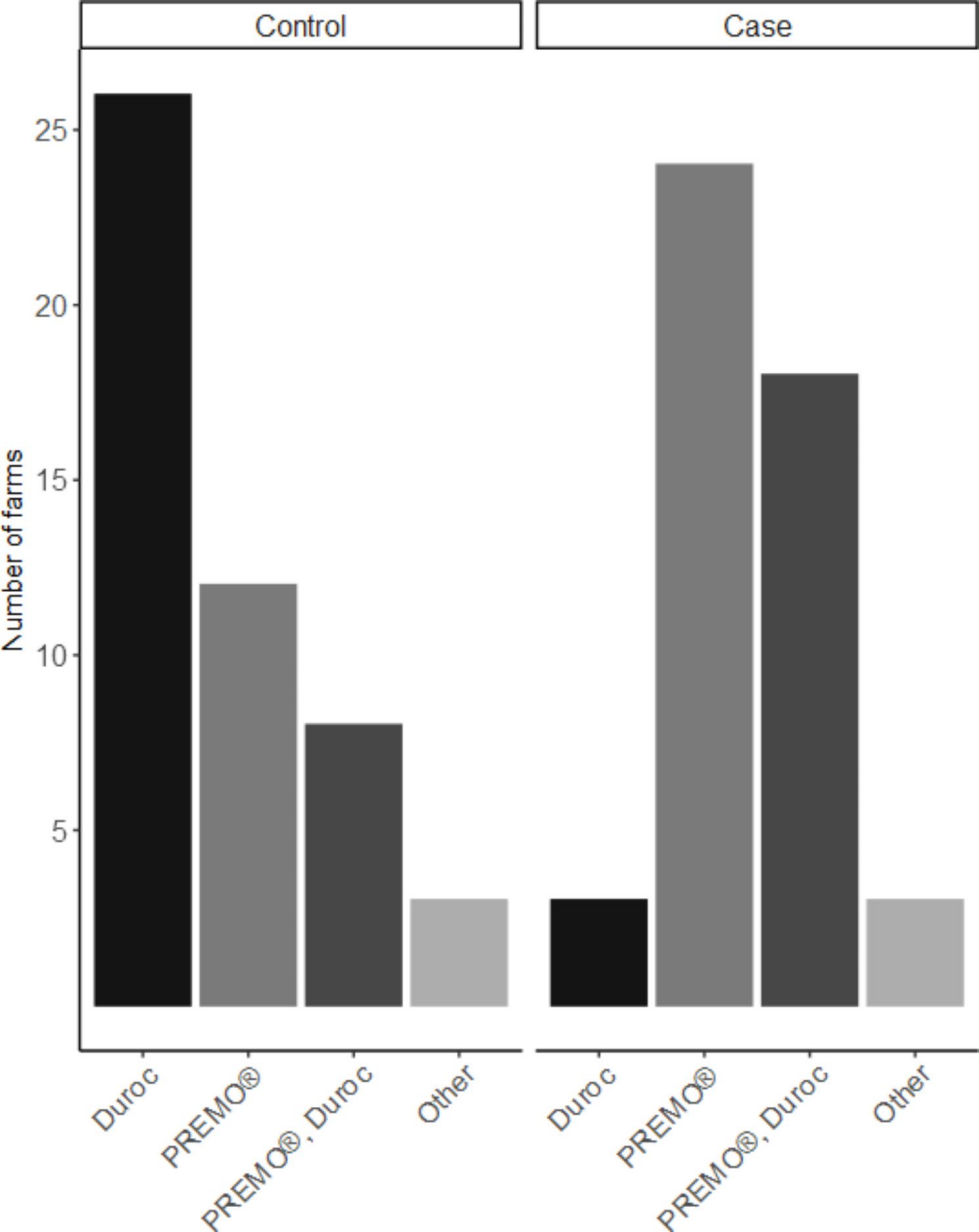
Distribution of sire breeds in HBS case and control farms. This figure is based on data of 48 case and 49 control herds

**Fig. 3:**
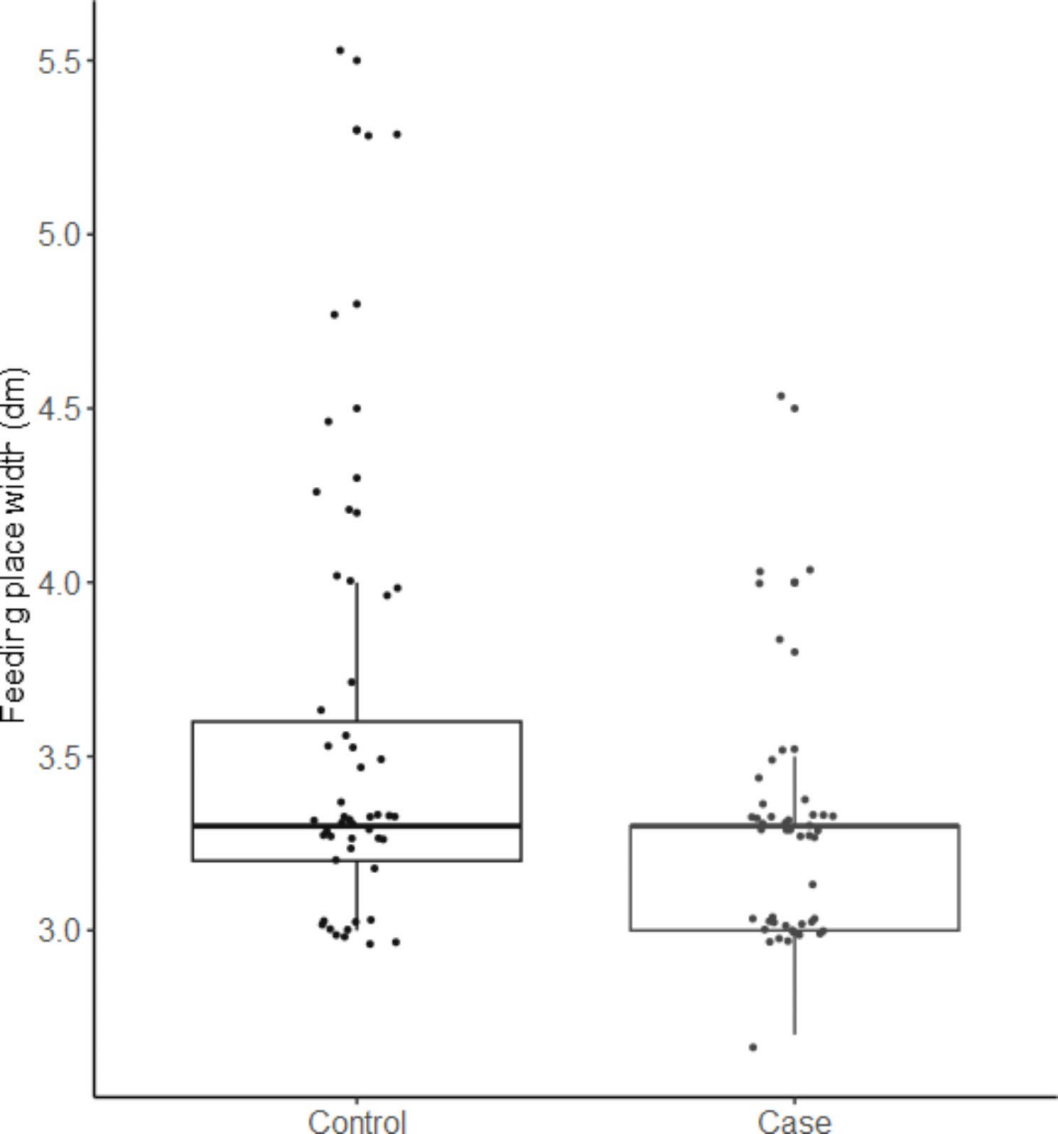
Feeding place width per finisher pig on HBS case and control farms. Control = 49 herds, Case = 48 herds, Dots = jitter plot, Bar = median

**Fig. 4:**
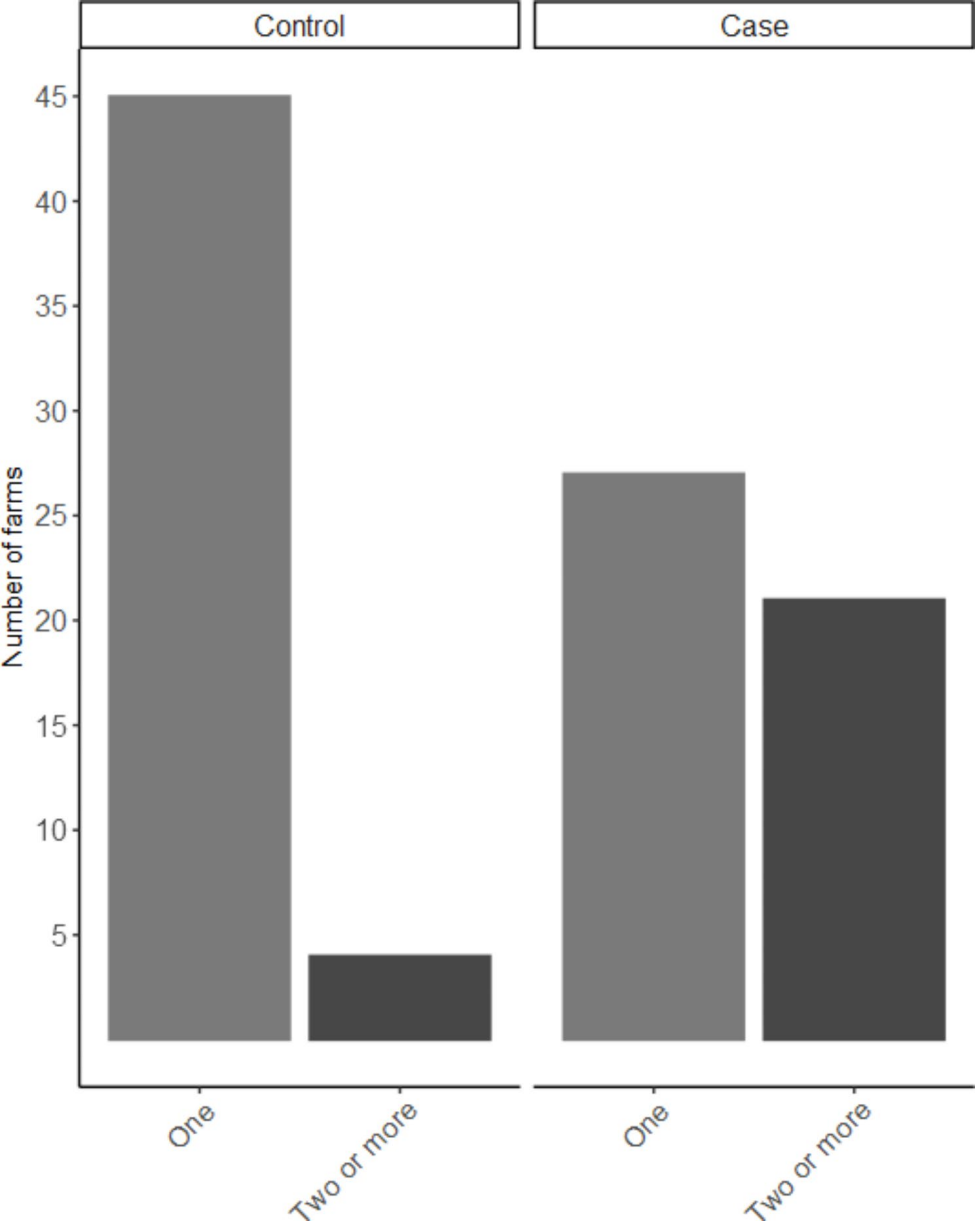
Number of origin of pigs per batch. This figure is based on data of 48 case and 49 control herds

**Fig. 5:**
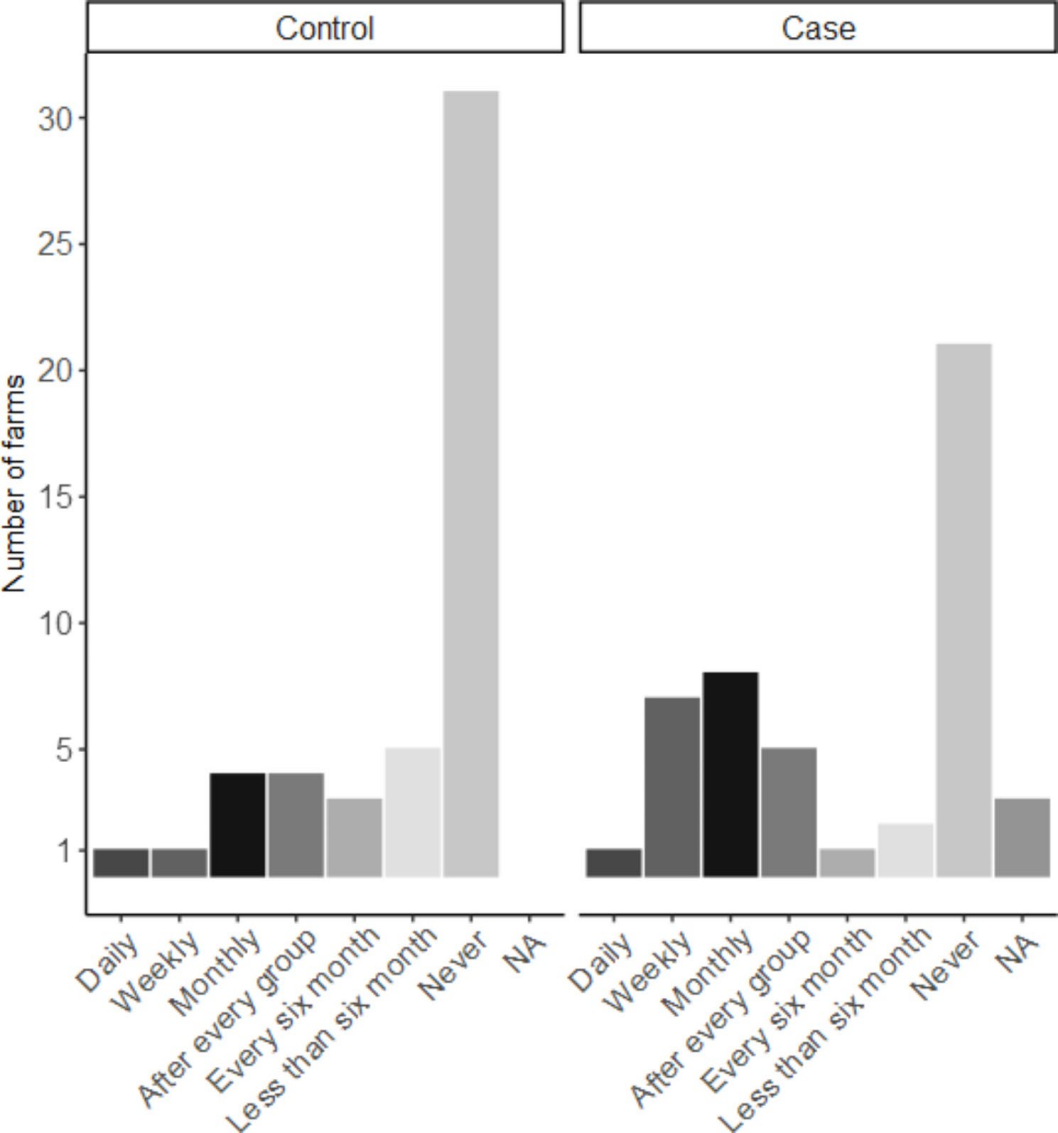
Frequency of cleaning of the distribution pipes. This figure is based on data of 48 case and 49 control herds

By means of questionnaire as well as measurements taken by the principle investigator of this study, a large number of variables were gathered. Only variables with a significant association (p-value ≤ 0.1) with the outcome (i.e. case or control farm) in the univariable model were described further (Tables 1 and 2). The geographical distribution of the farms visited across Switzerland is in agreement with the distribution of pig herds with finisher pigs in Switzerland (Fig. 1). Pigs were categorised into two groups by means of bodyweight, grower pigs with 25-60 kg bodyweight and finisher pigs with 60-120 kg bodyweight. In this study the mainly used breeds for meat production were considered, sire lines Large White (PREMO®), Duroc, Piétrain and dam lines Swiss Large White, Swiss Landrace (Fig. 2). Due to limited amount of Swiss Large White and Swiss Landrace dam lines as well as Piétrain sired pigs those breeds were grouped together as other. The feeding place width per pig was calculated for each herd in pens with finisher pigs and converted to decimetres (dm) for better understanding and comparison between case and control herds (Fig. 3). For the variable origins of pigs the answers were split into two categories, having all pigs per batch from one single origin or alternatively the pigs coming from two or more origins. Farmers breeding their own grower pigs were put in the one origin only category (Fig. 4). In this study the frequency of cleaning of the distribution pipes was divided into seven groups (i.e. never, less than every six months, every six months, after every group, monthly, weekly and daily) and the group never was chosen as comparison value (Fig. 5).

**Table 1:** Descriptive statistics of continuous variables with a p-value < 0.1 in the univariable statistical analysis (logistic regression). Results are sorted by case and control group

Case herds	n	Median	Min	Max	p-value
Pigs per group grower	48	30	8	80	**0.043**
Pigs per group finisher	48	25	6	80	0.083
Percentage of HBS mortality on total mortality	48	80	8	99	**< 0.001**
Feeding place width per pig in dm (finisher)	48	3.3	2.7	4.5	**0.018**
Minimal height of drinker in cm	48	40	13	55	**0.019**
Control herds					
Pigs per group grower	49	24	7	54	**0.043**
Pigs per group finisher	49	20	6	50	0.083
Percentage of HBS mortality on total mortality	49	20	1	98	**< 0.001**
Feeding place width per pig in dm (finisher)	49	3.3	3.0	5.5	**0.018**
Minimal height of drinker in cm	49	42	20	70	**0.019**

This table is based on data of 48 case and 49 control herds. Data was not normally distributed. *HBS*: Haemorrhagic bowel syndrome, *dm*: decimetre, *cm*: centimetre

**Table 2:** Descriptive statistic of categorical variables with a p-value < 0.1 in the univariable statistical analysis (logistic regression). Results are sorted by case and control group

Categorical variable	Overall	Case herds	Control herds	p-value
Breed of sire (%)				**< 0.001**
Duroc	22.68	4.16	40.82	
PREMO®	35.05	50	20.41	
PREMO® / Duroc	26.81	37.5	16.33	
other	15.46	8.34	22.44	
Origin of pigs per batch				**< 0.001**
One origin	74.23	56.25	91.84	
Two or more origins	25.77	43.75	8.16	
Frequency of stabulation				0.077
Weekly	11.34	18.75	4.08	
Every two weeks	37.11	37.5	36.73	
Every three weeks	13.41	8.34	18.37	
Monthly	11.34	6.25	16.33	
Other	26.8	16.16	24.49	
Frequency of cleaning of the distribution pipes				**0.035**
Daily	2.06	2.08	0	
Weekly	8.25	14.58	2.04	
Monthly	12.37	16.67	8.16	
After every group	9.28	10.42	8.16	
Every six month	4.12	2.08	6.12	
Less than six month	7.2	4.17	10.21	
Never	53.61	43.75	63.27	
NA	3.11	6.25	0	
Mode of cleaning of the distribution pipes				0.096
Acid solution	16.5	22.92	10.2	
Alkaline solution	16.5	20.83	12.25	
Other	57.72	50	65.31	
Frequency of cleaning of the storage containers				0.085
Daily	2.07	2.09	3.07	
Weekly	8.25	14.58	4.08	
Monthly	12.37	16.66	8.16	
After every group	9.28	10.42	8.16	
Every six months	4.12	2.09	3.07	
Less than six months	7.2	4.16	10.2	
Never	53.61	43.75	63.26	
NA	3.1	6.25	0	

This table is based on date of 48 case and 49 control herds. Data was not normally distributed. *NA* = no answer.

### Univariable analysis

Answers to 73 variables, gathered by questionnaire as well as own measurements, were tested individually in an univariable model against the outcome variable case vs. control farm as an intermediate step before multivariable analysis (Additional file 1 & 2). Eleven of the 73 variables showed an association (p ≤ 0.1) to the outcome variable. Correlation analysis revealed that none of those eleven variables were strongly correlated with each other (p < 0.6). An overview of the eleven parameters with the corresponding univariable p-values is presented in Tables 1 and 2.

### Multivariable analysis

Four of the eleven variables entered into the multivariable logistic regression model showed a significant influence on the outcome variable (p < 0.05). Breed was significantly associated with the farm being a HBS-case farm, with PREMO® sired pigs having the highest odds ratio (OR) of 147.5 compared to Duroc, followed by farms having other sire breeds (OR 67.8) and farms with PREMO® as well as the Duroc sired pigs in their fattening groups (OR 66.4). Having two or more origins of the pigs per group showed an OR of 52.1 for the outcome of case farm as compared to having one origin of pigs per group. As management factor, the frequency of cleaning of the distribution pipes showed a significant association with the outcome. Farms which cleaned the pipes weekly had a higher chance of being a case farm compared to farms with no cleaning ever (OR = 149). Feeding place width per finisher pig as a factor of pen design and management showed an OR of 0.071 for each 1 dm more width per finisher pig. Further information on the multivariable analysis is presented in Table 3. None of the risk factors significant in univariable analysis were relevant confounders (defined as changing the effect size of another risk factor by > 20%). Interactions between significant risk factors were not analysed because including interaction terms in the model resulted in small subgroups and poor model convergence.

**Table 3:** Risk factors for being a HBS case farm significant in multivariable logistic regression analysis

Risk factors	OR^1^	Lower^2^	Upper^3^
Sire breeds (compared to Duroc)			
PREMO®	147.53	8.19	2657.92
PREMO® / Duroc	66.41	4.3	1025.77
Other	67.76	2.47	1860.62
Origin of pigs per batch (compared to one origin)			
Two or more origins	52.12	4.28	635.16
Frequency of cleaning of the distribution pipes (compared to never)			
Daily	1.85	0.09	38.24
Weekly	149.02	3.05	7281.87
Monthly	7.42	0.4	136.01
After every group	0.94	0.09	9.96
Every six month	0.2	0.01	5.49
Less than six month	7.42	0.4	136.01
Feeding place width per finisher pig in dm			
Per 1 dm more	0.071	0.01	0.78

*This table is based on data of 48 case and 49 control herds*. ^*1*^*Odds ratio;*^*2*^*Lower 95% confidence limit;*^*3*^*Upper 95% confidence limit*

## Discussion

The aim of this study was to identify risk factor for the occurrence of HBS in Swiss finisher pigs with focus on environment and management factors as well as breed predisposition. Overall, 97 farms were investigated (48 case farms and 49 control farms), over a time period of 13 months, a representative sample population of Swiss finishing farms could be realised to find possible risk factors for the occurrence of HBS in grower-finisher pigs. Our results are unlikely to be affected by a selection bias because the farms were solely chosen based on their mortality rate caused through HBS. The time-consuming visits as well as the cooperation of the farmers to allow visits and invest their time for this project prevented a more sophisticated selection of farms and the inclusion of more farms. Furthermore, farms that had an HBS problem were not necessarily investigated when a HBS case occurred but rather having had a defined percentage of cases in the last 6 to 12 months. Environmental factors such as indoor and outdoor temperatures as well as air draft might have been different at the time of HBS cases.

After univariable analysis a total of eleven variables were significantly related to the outcome variable of being a HBS case farm and therefore put into a multivariable logistic regression model. The highest odds ratio (OR) was found for the variable pig breed, concerning the swine breeds that are found on each farm, followed by number of origins per pig group and frequency of cleaning of the distribution pipes and feed place width per animal (for finisher pigs).

A possible genetic component to HBS was suspected in different studies from the USA [4] and South Africa [14] attributing the higher frequency of HBS to the longer carcass of modern pigs. Since breeds used for meat production vary between countries in their genetic composition but also their frequency of use, no data about the correlation between Swiss pig breeds and the occurrence of HBS was available. Our study revealed a significant effect of PREMO® (Swiss Large White sire line) on the occurrence of HBS in comparison with Duroc. However, the OR of 147 should be treated cautiously because the breed specified by the farmer was not verified within the supply chain or with genetic markers and the 95% confidence interval is relatively large. Regardless, the lower boundary of the 95% confidence interval (OR = 8.19) still shows that using PREMO® is an important risk factor for HBS in Switzerland. A similar situation concerning OR and the 95% confidence interval was detected for farms having both PREMO® and Duroc in their stables. Due to lack of information on the breed of died pigs on mixed farm, no further conclusion can be drawn. However, it can be hypothesized that in mixed groups Duroc will limit the risk for HBS cases. A possible explanation for these findings could be the fact that the carcasses of PREMO® pigs are longer than those from other breeds such as Duroc and Piétrain. Furthermore, the PREMO® breed had been extensively selected for feed conversion ratio, average daily gain and lean meat percentage, although Duroc has even greater growth potential and Piétrain more lean meat than PREMO. These four characteristics (i.e. length of carcass, feed conversion ratio, average daily gain and lean meat percentage) combined could have an important effect on the digestive system as well as the abdominal structure and the available space in the abdominal cavity. These interactions could increase the risk for torsion of the intestines and death due to HBS. Another speculation is, that modern pigs in fattening systems have reached high level of performance and are therefore running at their possible limit. Minor changes in their normal environment and feeding practices can overstrain the performance of said pigs and therefore lead to problems such as HBS. The Swiss AI market already reacted to the higher HBS risk of PREMO. In the last two years the number of artificial inseminations with semen from Duroc boars in Switzerland significantly increased (almost doubled) [17].

The feeding space of pigs was detected as a risk factor for HBS in comparison to other studies neglecting to investigate this factor of animal housing [5, 18]. In the present study an additional of 1 dm of feed place width per finisher pig was linked to a decreased risk of HBS with an OR of 14.1. It might be that less space increases the stress level of pigs at feeding time, leading to the assumption that stress at feeding time as well as directly after has a significant impact on HBS occurrence. If feeding space is limited a competition over feed will be established, even if enough feed is present, stronger pigs having more access but still needing to defend their space whereas weak pigs will eat as fast as possible to get enough feed once they are at the feeding trough. This stressful behaviour can lead to more air being swallowed while feeding and therefore leading to more air in the gastrointestinal tract. Furthermore, the increased activity provoked by the stressed behaviour can cause pigs to try and go under other pigs, lifting them up and disrupting the normal feed intake as well as compressing and decompressing the abdomen in fast succession. Movement combined with gas in the stomach and intestines can increase the risk for torsion of intestines in the abdomen along the mesenteric root. Leading to the question if the minimal standard width per finisher pig should be altered to reduce stress at feeding. The minimal standard feeding space width per pig for Swiss fattening pigs (i.e. 30 cm/pig) is comparable to the ones from Sweden (i.e. 31 cm/pig). Studies from both countries from Sweden as well as from the USA have not been able to identify the feeding space width as a risk factor for HBS but was acknowledged in both studies to have a significant impact on the duration of feed intake and the activity at feeding time [5, 19]. Furthermore, the study from Sweden focuses on aggression and corresponding lesions and not causes for mortality in investigated herds.

The number of origins per batch of pigs has not yet been described in context of HBS. Rather the influence of multiple origins per batch in relation to infectious agents as well as the transmission of such was investigated [20]. In the present study the chance to be a HBS case farm is 52 times more likely if two or more origins of pigs were given per batch as compared to having one origin only. Therefore, an infectious component to the HBS complex cannot be excluded, and investigation into infectious agents relevant to the fattening age group transmitted through faecal material should be conducted. As non-infectious causes the genetic similarity and the comparable microbiome of pigs being born in the same farm from the same sire (breed) could explain the connection found between HBS and number of origins. Furthermore, the social hierarchy of pigs is very strong leading to fights when new pigs, at the time of arrival on the farm, are mixed together, the same behaviour can be seen if pens are regularly mixed together instead of incorporating an all-in-all-out system [19, 21]. Competition at feeding time or at the feeding station can be strong in socially unstable groups leading to more stress and higher activity and might therefore be a promoting risk factor to torsion of the intestines and deaths due to HBS.

The importance of feed distribution system hygiene was described in multiple studies [3, 8, 9]. Therefore, a special focus was laid on the cleaning management, especially cleaning frequency and the used methods, of the feed distribution systems as well as feed storage containers. The used methods on the farms were investigated by multiple studies but none of those studies investigated the frequency of cleaning. Interestingly, farms which practice cleaning of the distribution pipes every six months or after every group were slightly less likely to be HBS case farms than farms with no cleaning. However, farms with a higher cleaning frequency (weekly, monthly and daily) had higher odds of being HBS case farms than farms with no cleaning. Therefore, it might be that frequent cleaning leads to an imbalance of the microflora in the feed distribution system [22]. Hence, the building of a new microflora in the feeding systems favours the growth of coliform bacteria until an adequate lactic acid bacteria flora is established [28], which might lead to a high number of coliform bacteria in the feed. Therefore, the cleaning interval of the feed distribution system can improve the feed hygiene and might reduce HBS on herd level. Furthermore, one study claimed, that the incubation of the freshly cleaned pipes with a lactic acid bacteria strain could improve the establishment of an adequate flora and therefore shortens the time of coliform overgrowth in the feed distribution system [22]. This effect could not be proven with our questionnaire, because none of the farms used an incubation strain after cleaning the feeding system. However, no significant differences between the cleaning methods (i.e. acid solution, alkaline solution, combination of acid and alkaline solution, water and barley corns) of the feed distribution system on the HBS prevalence could be detected.

Our study did not confirm previously described risk factors for HBS including liquid feeding systems [6] whey [2, 15], routine use of antibiotics [8, 9] as well as sex of the pigs [3, 16] and seasonal effect [3, 4]. Although, liquid feeding systems were used in 60.8% of all investigated farms, no statistically significant difference between case and control farms on the prevalence of HBS could be detected. In addition, including whey as component of the diet is discussed as a risk factor in multiple studies [8, 9, 12, 15] and often mentioned by practitioners, but showed no significant effect in this study. However only the usage of whey in the diet was asked by questionnaire, and therefore, no analysis of the quality or bacterial contamination of the whey was conducted. Hence, no valid conclusion can be drawn about the effect of whey on HBS, but warrants that further research is needed to exclude whey as a potential risk factor. Since the routine use of antibiotics, for example as treatment at arrival of a new group, is not allowed in Switzerland the effect of such an application could not be examined. Reason for the disallowance of routine antimicrobial use in Switzerland is the prudent use of antibiotics act aiming to reduce the bacterial resistance situation. In addition, a controversial gender effect was described for HBS revealing a higher prevalence in female pigs in a Swiss study [3] compared to a higher prevalence for castrated males and boars in a study from Great Britain [16]. In the questionnaire no information about the gender effect was available, because the farmers did not take notes of the diseased pigs’ gender. Furthermore, no seasonal effect on the occurrence of HBS could be detected in this study in comparison with an older study from Switzerland [3]. A possible explanation for this difference in findings could be the generally better stable climate and management found in fattening stables today as compared to the older study [3]. Differences in temperature and humidity can be regulated better with modern climate installations in pig stables leading to a more stable climate and therefore reduces stress in pigs. Knowledge about the tolerable difference in day and night temperature is increased and modern fattening stables often use micro climate zones to further accommodate pigs better.

## Conclusion

The present study is one of the first case-control studies looking categorically for risk factors for the occurrence of HBS in grower-fattening pig herds. This study revealed four risk factors including sire breed PREMO®, number of origins of pigs per batch, frequency of cleaning of the feed distribution system and feeding place width per animal (finisher pig).

These results corroborate that HBS has multiple risk factors that cannot solely be attributed to one management or housing category. Moreover, the factors individually should not be taken as one single cause for HBS, but rather be interpreted as factors increasing the risk of HBS and jointly contribute to increase said risk without influencing each other directly. The four risk factors may be considered to reduce disease incidence in farms with high mortality through HBS. Furthermore, a thorough investigation of the individual farm with inclusion of the presented risk factors is important to improve the HBS problematic on the farms. Because it is a multifactorial disease-complex a farm- individual solution should be sought, a general solution for fattening farms is not possible and should not be promoted without previous investigation of the farms.

A good cooperation between farmers, veterinarians and marketers is important to analyse and improve the risk factors found in this study in herds with HBS problems. The importance of maintaining the optimal cleaning frequency and finding the optimal feeding place width for the individual conditions on the farms must be supported by the veterinarian. Whenever possible, it is preferable to source all animals in a group from a single herd of origin; this also brings advantages in terms of biosecurity and must be supported by the different stakeholders.

Further studies need to be realised to find possible risk factors not grasped in this study, for example the importance of feed quality, feed composition and presence of intestinal pathogens. Also, studies aiming at identifying the genetic background of HBS should be done.

## Material & methods

### Ethical issues

The protocol of this study was approved by the responsible Cantonal Veterinary Office of Berne (license no. BE48/2021; 33,799).

### Study design and herd selection

This case-control study included 100 Swiss pig herds (50 case farms and 50 control farms). This sample size ensures a power of 80% to detect odds ratios larger than 3.5 at the 5% significance level [23]. Inclusion criteria for HBS farms was a mortality rate caused by HBS of 1.5% o more, and a herd size with 600 or more slaughtered pigs per year. Control herds had a mortality rate caused by HBS of 0.25% or less with the same number of slaughtered pigs per year. The total mortality rate of the herd (i.e. HBS as cause of death as well as any other disease) was not considered a criteria for the farms. Herds were selected using the database records of the SUISAG Swiss Pig Production, department for Health Service (SGD) including data from 490 000 fatting places. The following parameters were used to select the farms: herd size, health status, mortality rate and reason (recorded in an electronic journal), as well as the electronic treatment journal (EBJ). Selected farms were chosen in two steps, firstly contacted by SUISAG to ask for permission to pass on contact information to the principle investigator of this study. Secondly, the eligibility of the farms were controlled by either herd vets, marketers or both. After permission and controlling the eligibility, the principle investigator contacted the farms and verified the mortality rate and causes of death in the herd. Furthermore, the farmers received a detailed information about the study design and the planned investigations on the farm. At the herd investigation, each farmer signed an agreement for the use of the data and samples gathered at his/her farm as well as a declaration of freedom of highly contagious disease. All data were anonymised before further analysis. Herds that had no detailed recordings of the mortality rate were excluded. In addition, no management changes on housing, feed or breed of the finisher pigs had to have occurred in the least 6 months before the examination of the herd.

Visits to the farms were performed between September 2021 and October 2022 by the principal investigator with no preference of season or time of day.

### Questionnaire

Questions were created after a thorough literature research and were validated on three farms not included in this study (two HBS and one control herd). The validated questionnaire was created with the LimeSurvey online tool (LimeSurvey Cloud Version 3.28.7). The questionnaire was conducted by the principal investigator of the present study on the farm together with the farmer. The question types included multiple choice, single choice as well as free text answers based on the range of expected answers and biological and statistical reasonability.

The questionnaire included 88 questions of which the first part covered general information about the herd, housing and caretaking of the pigs as well as management and hygiene of the pens and feeding systems, and lastly performance data. The second part investigated the occurrence of HBS in the herd. For herds without HBS cases in the last 6 to 12 months these questions were not asked. The time period of the questionnaire included only the last 12 months to reduce recall bias. In addition to the questionnaire, an investigation of the pig pen was done to gather subjective parameters as well as measurements of pen size, number of pigs per pen, water flow rate, number of drinkers, height of drinkers, water pH, length of feeding trough, feed pH, air temperature and air draught. After the investigation, the pig density as well as the number of feeding spaces, feeding space width per pig and number of pigs per drinker were calculated. For the number of feeding spaces per pen the minimal width of the Swiss animal protection law was used (i.e. 2.7 dm for pigs < 60 kg bodyweight and 3.0 dm for pigs ≥ 60 kg bodyweight), giving the maximally allowed feeding places for the measured length of the feeding trough.

### Data processing and statistical data analysis

The answers from the questionnaire were gathered via the online survey tool LimeSurvey (LimeSurvey Cloud Version 3.28.7), subsequently transformed in an Excel table (Microsoft Excel, 2010), visually checked for completeness, and imported to the statistical software R (R version 4.2.1) for descriptive analysis and visualization. Measurements taken on farms were transferred to the same Excel table as the answers from the questionnaire and treated the same as the answers in the table. Variables with multiple categories that had a small number of answers were combined if biologically reasonable, for example less frequent pig breeds (i.e. Hampshire, Piétrain and pure maternal lineage Large White and Swiss Landrace) were summarised in the group “others”, for continuous variables, a categorisation was done using known cut-off values if such values were available or otherwise according to the distribution after descriptive statistics were realised. For the variable origins of pigs the answers were split into two categories, having all pigs per batch from one single origin or alternatively the pigs coming from two or more origins. Farmers breeding their own grower pigs were put in the one origin only category. In this study the frequency of cleaning was divided into seven groups (i.e. never, less than every six months, every six months, after every group, monthly, weekly and daily) and the group never was chosen as comparison value. To prevent problems with collinearity in the regression model, potential risk factors for HBS were checked for correlation with each other by cross tabulations and phi coefficient (phi cut-off value: 0.6). The univariable as well as the multivariable models were computed using the statistical software NCSS (NCSS 2022). The outcome variable of each model was being a HBS case farm yes or no and therefore being treated as binary variable with exclusive categories. Initially, potential risk factors for being a case farm were screened in univariable logistic regression models. Only variables that were associated with the outcome (p < 0.1) in the univariable logistic regression model were kept and entered into the multivariable logistic regression model. It was checked whether confounding occurred by evaluating whether removing a variable changed the coefficient of another risk factor by more than 20%. Variable selection was performed by stepwise backward selection. To check whether the final model was stable, a stepwise forward selection strategy was also tried, which resulted in the same model as the stepwise backward selection. The level of significance was set at p < 0.05.

Geographical distribution of the examined herds was visualised using QGIS (www.qgis.org version 3.28.3).

## Data Availability

The dataset for the risk factor analysis study is available from the corresponding author on reasonable request.
